# Validity and reliability evaluation of the Functional Assertiveness Scale for community pharmacists: a cross-sectional study

**DOI:** 10.1186/s40780-026-00609-8

**Published:** 2026-07-15

**Authors:** Mitsuaki Ishii, Sachiko Ozone, Shoichi Masumoto, Reiko Mizutani, Toshinari Mitsuoka

**Affiliations:** 1 Department of Pharmacotherapy, Faculty of Pharmaceutical Sciences, Hokkaido University of Science, 15-4-1, Maeda 7-jo, Teine-ku, Sapporo, Hokkaido 006-8585 Japan; 2 Department of Family Medicine, General Practice and Community Health, Institute of Medicine, University of Tsukuba, 1-1-1, Tennodai, Tsukuba, Ibaraki 305-8575 Japan

**Keywords:** Functional assertiveness, Community pharmacists, Psychometrics, Questionnaire

## Abstract

**Background:**

Interprofessional collaboration between physicians and pharmacists is important for ensuring medication safety. Community pharmacists play a vital role in this process through prescription recommendations to physicians. Assertiveness, defined as a communication style that attempts to enhance mutual understanding while respecting both oneself and others, is recognized as useful for making prescription recommendations. Based on the concept of assertiveness, “functional assertiveness,” which focuses on achieving tasks while maintaining interpersonal relationships, has been proposed and the Functional Assertiveness Scale (FAS) was developed to evaluate it. Although functional assertiveness has been reported to be useful for achieving the task of ensuring medication safety while maintaining professional relationships with physicians, its applicability to community pharmacists has not been verified. This study aimed to investigate the reliability and validity of the FAS for Japanese community pharmacists and evaluate its utility.

**Methods:**

A cross-sectional study was conducted using an online questionnaire targeting 2,190 community pharmacists between July and September 2025. Structural validity was examined using exploratory factor analysis (EFA) and confirmatory factor analysis (CFA). Internal consistency was assessed using Cronbach’s α coefficient. Construct validity was verified by calculating Spearman’s rank correlation coefficients between FAS scores and the Assertive Self-expression (AS) score of the Interprofessional Assertiveness Scale.

**Results:**

Analysis was performed on 415 participants (mean age 41.0 years; 54.9% women). EFA confirmed a two-factor structure consistent with the original scale. CFA showed good model fit based on SRMR (0.048); however, CFI (0.893) and TLI (0.867) were slightly below conventional thresholds. Cronbach’s α for the total FAS was 0.85, indicating good internal consistency. Construct validity was supported by a significant positive correlation between FAS total score and AS score (rho = 0.34, *p* < 0.01).

**Conclusions:**

FAS demonstrated sufficient reliability and a clear two-factor structure for application among Japanese community pharmacists. Our findings suggest that FAS serves as a useful tool for objectively measuring functional assertiveness among community pharmacists. On the other hand, the CFA results indicate the need for future item optimization specifically tailored to pharmacy practice. Further studies are needed to develop a pharmacist-specific version to contribute to evaluating educational programs and enhance interpersonal communication in pharmacy practice.

**Supplementary Information:**

The online version contains supplementary material available at https://doi.org/10.1186/s40780-026-00609-8.

## Introduction

Interprofessional collaboration between healthcare professionals is essential for providing high-quality, safe patient care [1]. Pharmacotherapy is a core component of patient management, and collaboration among professionals is essential to prevent the occurrence of avoidable adverse drug events [2]. Pharmacists play an important role in preventing adverse events by collaborating with physicians when concerns about medication safety arise [3]. A previous study reported that physician-pharmacist collaboration prevents drug-drug interactions and duplicate prescriptions [4]. The importance of communication between physicians and pharmacists as the core of collaboration is increasingly recognized.

Although the importance of communication is well-established, its effectiveness in clinical practice is influenced by how it is executed. In interactions between healthcare professionals, a previous study reported differences in preferred communication styles [5]. Pharmacists exhibit three distinct styles in their interactions with physicians: avoidant, assertive, and cooperative [6]. Physicians appreciate pharmacists’ prescription proposals [7], even though they may reject or express negative feelings to them [8]. This suggests that the acceptance or rejection of prescription proposals is not solely determined by the appropriateness or medical accuracy of the proposal, but is also influenced by communication style. Therefore, to promote proactive proposals from pharmacists to physicians, it is necessary to focus not only on reviewing prescription details but also on enhancing the pharmacists’ own communication skills.

Assertiveness is a necessary communication skill for healthcare professionals. Assertiveness is defined as a communication style for expressing one’s own thoughts and desires openly while respecting others. It is a skill that can be acquired [9, 10] and measured [11–14]. Assertiveness is defined as a style of self-expression that demonstrates consideration for both oneself and others with the aim of enhancing mutual understanding, which is distinct from aggressive expression [15, 16]. This approach is expected to be utilized more extensively as a tool for improving interpersonal communication skills in the field of patient safety [17]. In community pharmacy settings, higher levels of assertiveness among pharmacists are associated with a higher frequency of pharmacist-initiated prescription changes [18] and experience with pharmacist-led deprescribing [19]. Thus, establishing methods to accurately assess and enhance pharmacists’ assertiveness is essential for advancing clinical pharmacy practice.

Despite the importance of assertiveness being recognized, its measurement remains challenging. In defining assertiveness, three styles of self-expression have been identified: nonassertive, aggressive, and assertive. Assertiveness assessment scales generally evaluate the degree of these three forms of self-expression [12, 14, 20]. However, in real-world communication, these self-expression styles are applied in complex ways, and the distinction between aggressiveness and assertiveness is often unclear [21]. To address these challenges, functional assertiveness was proposed as an assertive self-expression style that promotes effective task achievement in difficult situations where the speaker must achieve specific goals, while also being perceived as communicating in an appropriate way by the listener [21]. Mitamura et al. developed a 12-item Functional Assertiveness Scale (FAS) to evaluate this concept [21] The FAS consists of two subscales: Objective Effectiveness (OE) and Pragmatic Politeness (PP). Its validity and reliability have been examined among university students, and its use was expanded to the parents of children with developmental disabilities [22] and psychiatric patients [23]. The role of pharmacists in recommending improvements to physicians to enhance medication safety correspond to the task achievement contexts defined by the FAS. A previous study reported that greater functional assertiveness among community pharmacists in their communication with physicians was associated with lower levels of stress related to deprescribing recommendations [24]. However, the validity and reliability of the FAS within the pharmacist population remain to be elucidated. There is a need for tools that can objectively evaluate individual skills in the specific communication setting of prescription recommendations for pharmacists. If the FAS is shown to be applicable to community pharmacists, it could become a useful tool for measuring communication strategies that contribute to medication safety as well as being utilized to evaluate future communication education programs. The aim of this study was to psychometrically verify the reliability and validity of the FAS among community pharmacists working in Japan.

## Methods

### Research design and participants

A cross-sectional study was conducted using anonymous self-administered questionnaires targeting community pharmacists. The participants were pharmacists who belonged to pharmacies that agreed to cooperate in the study and who routinely dispensed prescriptions as part of their daily work. This study was conducted at two pharmacy chains: a nationwide chain and a chain operating in Hokkaido, an island in northern Japan.

### Data collection

Participants were invited to participate in the survey via email through their respective company representatives. The online survey was conducted at two pharmacy chains for one month each between 1 July and 30 September 2025. Participants were invited to the survey site, Survey Monkey^®^, via a URL or QR code attached to the invitation email. The questionnaire form website was accessible without a password, so respondents were instructed to submit their answers only once. Answering all questions was mandatory. To prevent duplicate responses from the same person, the last four digits of the respondent’s date of birth and the last four digits of their phone number were collected as a registration number, and in the event of duplicate access, responses after the first attempt were invalidated.

### Sample size calculations

The sample size was determined based on the Consensus-based Standards for the Selection of Health Status Measurement Instruments (COSMIN) [25] as 10 responses per item. As the FAS is a 12-item scale; therefore, at least 120 responses were required. Estimating an online survey response rate of approximately 20%, it was calculated that a minimum of 600 participants would be necessary.

### Measurement items

The FAS is a self-report measure comprising a total of 12 items, based on previous research [21]. The scale consists of two subscales: OE (six items) and PP (six items). Each item was rated on a five-point Likert scale from 1 = *Always disagree* to 5 = *Always agree*. Total FAS score (range: 12–60) and total score for each subscale (each range: 6–30) were calculated from the sum of the scores for each item. A higher score indicates greater characteristics of functional assertiveness. The Interprofessional Assertiveness Scale (IAS) was used to assess the construct validity of the FAS. The IAS is an 18-item questionnaire assessing three self-expressions (non-assertive, assertive, aggressive) within interprofessional teams. In this study, only the Assertive Self-expression (AS) domain (seven items) of the IAS was used to verify construct validity [14]. Responses were assessed using a 6-point Likert scale from 1 = *Not applicable at all* to 6 = *Very applicable*. IAS-AS domain scores ranged from 7 to 42, with higher scores indicating higher levels of assertive self-expression. Participants’ demographic characteristics included age, gender, employment status, years of experience as a pharmacist, managerial experience, education level, and participation in an assertiveness training program (including general hospitality training or assertiveness programs for healthcare professionals). In addition, the evaluated related information, including participation in interprofessional care conferences, was defined based on the requirements of the Community-based Collaboration System Premium (*Chiiki-shien-taisei-kasan*), as stipulated in the Establishment of Facility Standards for Specially Designated Medical Fees [26].

### Evaluation of psychometric properties

In this study, validity and reliability were examined with reference to COSMIN [25]. The attributes of participants were described, and FAS scores, subscale scores, and AS scores were calculated. Regarding FAS responses, the ceiling effect and floor effect were examined by confirming their distribution. Ceiling and floor effects were deemed to be present when the distribution of the highest or lowest scores for each item exceeded 15% [27].

Structural validity was verified through a two-stage approach combining exploratory factor analysis (EFA) and confirmatory factor analysis (CFA). Based on the model presented in previous research, the factor structure of the FAS was explored using principal axis factoring (PAF) and Promax rotation. To assess the adequacy of sampling, the Kaiser-Meyer-Olkin (KMO) measure and Bartlett’s sphericity test were used. KMO was determined as follows: < 0.5 insufficient, 0.5–0.7 moderate, 0.7–0.8 good, 0.8–0.9 very good, > 0.9 excellent [28]. Bartlett’s test of sphericity was performed to assess significance and if significant, the data were considered appropriate for analysis [29]. The number of factors was determined based on a scree plot and factors with eigenvalues exceeding 1.0. Items with factor loadings of 0.30 or higher were adopted as acceptable. CFA examined whether the model fits the two-factor structure (OE and PP) proposed in a previous study [21]. Model fit was assessed using the comparative fit indices (CFI > 0.95), Tucker-Lewis index (TLI > 0.95), root mean square error of approximation (RMSEA < 0.06), and standardized root mean squared residual (SRMR < 0.08) as criteria indicating good fit [30]. Composite reliability (CR) and average variance extracted (AVE) were calculated to evaluate the reliability and convergent validity of the *factor* structure, based on the recommended cut-offs of > 0.7 for CR and > 0.5 for AVE [31]. To ensure the robustness of the factor structure and avoid overfitting due to the use of the same dataset, supplementary cross-validation was performed using a randomized split-sample method. The entire sample (*n* = 415) was randomly divided into two subgroups. EFA was performed on one subgroup (*n* = 196) and CFA was performed on the other (*n* = 219).

Reliability was verified by calculating Cronbach’s alpha coefficient to assess the internal consistency of the scale. A Cronbach’s alpha coefficient between 0.70 and 0.95 was determined to indicate sufficient reliability [27]. Item-total correlations were calculated to assess the degree of consistency between items and the total scale. A correlation coefficient > 0.2 was determined to indicate sufficient reliability [32].

To verify construct and criterion-related validity, the FAS was validated as an appropriate measure of assertiveness by calculating Pearson or Spearman correlation coefficients according to score distribution, and by examining whether positive correlations existed between FAS total score, each subscale score, and IAS-AS score. To verify criterion validity, a comparison of score distributions based on participation in assertiveness training was conducted. In addition, differences in score distributions based on participation in interprofessional care conferences, which showed distribution differences in a previous study [33], were examined using the Mann-Whitney U test.

All statistical analyses were conducted using IBM SPSS version 30.0 (IBM Corp, Armonk, NY, USA), and IBM SPSS Amos version 30.0 (IBM Corp, Armonk, NY, USA). Significance was defined as < 0.05.

### Ethical considerations

The study was conducted in accordance with the Declaration of Helsinki and approved by the Ethics Committee of Hokkaido University of Science (No. 25–06). All participants participated in the survey on a voluntary, anonymous basis, and were informed in advance that their responses to the survey were considered acceptance of their participation.

## Results

### Characteristics of participants

Of the 2,190 people invited to participate in the survey, 419 accessed the survey form site and responded. After excluding three individuals who provided identical “1” responses for all items and one individual whose reported education and age were inconsistent, data from 415 participants (18.9%) were analyzed. The mean age (standard deviation; SD) of participants was 41.0 years (11.0), and 54.9% were women (Table 1). Additionally, 21.9% of participants reported having participated in assertiveness training, and 59.5% had experience attending interprofessional care conferences.

**Table 1:** Participant characteristics (n = 415)

Characteristic	
Age, years, mean (SD)	41.0 (11.0)
Gender, n (%)	
Men	177 (42.7)
Women	228 (54.9)
Prefer not to respond	10 (2.4)
Employment status, n (%)	
Full-time	351 (84.6)
Part-time	64 (15.4)
Years of pharmacist experience, median (IQR)	13 (6–22)
Managerial experience, n (%)	
Currently a manager	133 (32.0)
Not currently a manager, but has past experience	96 (23.1)
No management experience	186 (44.8)
Education, n (%)	
Bachelor (4 years)	189 (45.5)
Bachelor (6 years)	199 (48.0)
Master’s degree	27 (6.5)
Participation in assertiveness training program, n (%)	
Yes	91 (21.9)
No	324 (78.1)
Participation in interprofessional care conferences, n (%)	
Yes	247 (59.5)
No	168 (40.5)

IQR, Interquartile range

### Distribution of responses, floor and ceiling effect

Table 2 presents the distribution of FAS responses and the mean and median scores. No subscales or total scores exceeded the preset criteria (15%) for floor or ceiling effects, indicating that the scale appropriately captured the variability in the participants’ responses.

**Table 2:** Distribution of Functional Assertiveness Scale (FAS) (*n* = 415)

	Item	Always disagree *n* (%)	Relatively disagree *n* (%)	Neutral *n* (%)	Relatively agree *n* (%)	Always agree *n* (%)	Mean (SD)	Medican (IQR)	Floor effect^a^ *n* (%)	Ceiling effect^b^ *n* (%)
FAS							36.72 (5.81)	37 (33–41)	2 (0.5)	0 (0.0)
OE							17.05 (3.90)	18 (14–20)	6 (1.4)	1 (0.2)
FAS1	I can get a teammate or a peer to change his/her behavior if the person is being disruptive.	32 (7.7)	130 (31.3)	195 (47.0)	57 (13.7)	1 (0.2)	2.67 (0.82)			
FAS2	I can get people to understand my own ideas even if my ideas are different from theirs.	11 (2.7)	90 (21.7)	208 (50.1)	101 (24.3)	5 (1.2)	3.00 (0.78)			
FAS3	I can get a person to improve his/her manners if I feel the person’s manners are not OK.	22 (5.3)	121 (29.2)	191 (46.0)	79 (19.0)	2 (0.5)	2.80 (0.82)			
FAS4	I can get a person to adjust his/her way of dressing if I feel the person’s appearance is not OK.	24 (5.8)	118 (28.4)	186 (44.8)	83 (20.0)	4 (1.0)	2.82 (0.85)			
FAS5	I can get a person to understand that he or she is being unjust if he or she points out my failures due to a misunderstanding.	31 (7.5)	128 (30.8)	164 (39.5)	84 (20.2)	8 (1.9)	2.78 (0.92)			
FAS6	I can get my friends to stop their annoying or troublesome actions.	20 (4.8)	100 (24.1)	171 (41.2)	119 (28.7)	5 (1.2)	2.97 (0.88)			
PP							19.67 (3.52)	20 (18–22)	2 (0.5)	1 (0.2)
FAS7	I don’t offend a disruptive teammate or a peer when I try to get that person to change his/her behavior.	12 (2.9)	73 (17.6)	218 (52.5)	106 (25.5)	6 (1.4)	3.05 (0.78)			
FAS8	I never make people feel bad when I try to get them to understand my own ideas.	8 (1.9)	65 (15.7)	237 (57.1)	103 (24.8)	2 (0.5)	3.06 (0.71)			
FAS9	I don’t needlessly embarrass someone when I try to get that person to improve his/her manners.	6 (1.4)	36 (8.7)	197 (47.5)	163 (39.3)	13 (3.1)	3.34 (0.74)			
FAS10	I cause minimal embarrassment to a person when I try to get that person to change the way he or she dresses.	4 (1.0)	29 (7.0)	182 (43.9)	184 (44.3)	16 (3.9)	3.43 (0.72)			
FAS11	I don’t get on a person’s nerves more than necessary when I try to get the person to understand that he/she is being unjust in pointing out my failures.	8 (1.9)	51 (12.3)	172 (41.4)	164 (39.5)	20 (4.8)	3.33 (0.82)			
FAS12	I don’t carelessly insult my friend when I try to get him or her to stop annoying or troublesome actions.	3 (0.7)	26 (6.3)	188 (45.3)	177 (42.7)	21 (5.1)	3.45 (0.72)			

^a^Defined as > 15% of participants having the lowest possible factor score.

^b^Defined as > 15% of participants having the highest possible factor score

FAS, Functional assertiveness scale; IQR, Interquatile range; OE, Objective effectiveness; PP, Pragmatic politeness; SD, Standard Deviation

### Verification of psychometric properties

#### Structural validity

For EFA, sampling adequacy (KMO statistic = 0.84) and suitability for factor analysis (Bartlett’s test of sphericity, *p* < 0.01) were confirmed, indicating that the data were appropriate for analysis. Using the PAF method, two-factor structure was confirmed as appropriate. Items with factor loadings > 0.3 obtained through Promax rotation were adopted for each factor (Table 3).

**Table 3:** Psychometric properties of FAS (*n* = 415)

	Factor loading	Internal consistency^a^	Composite Reliability	Average Variance Extracted
FAS		**0.85**		
OE		**0.86**	0.86	0.52
FAS1	0.73	0.67		
FAS2	0.60	0.59		
FAS3	0.83	0.73		
FAS4	0.83	0.73		
FAS5	0.61	0.57		
FAS6	0.67	0.65		
PP		**0.87**	0.87	0.54
FAS7	0.55	0.57		
FAS8	0.56	0.58		
FAS9	0.86	0.76		
FAS10	0.83	0.74		
FAS11	0.76	0.69		
FAS12	0.80	0.73		

^a^Assessed using Cronbach’s alpha for factor scores (shown in bold) and item–total correlation coefficients (Spearman’s rank correlation) for individual items

FAS, Functional assertiveness scale; OE, Objective effectiveness; PP, Pragmatic politeness

**Fig. 1:**
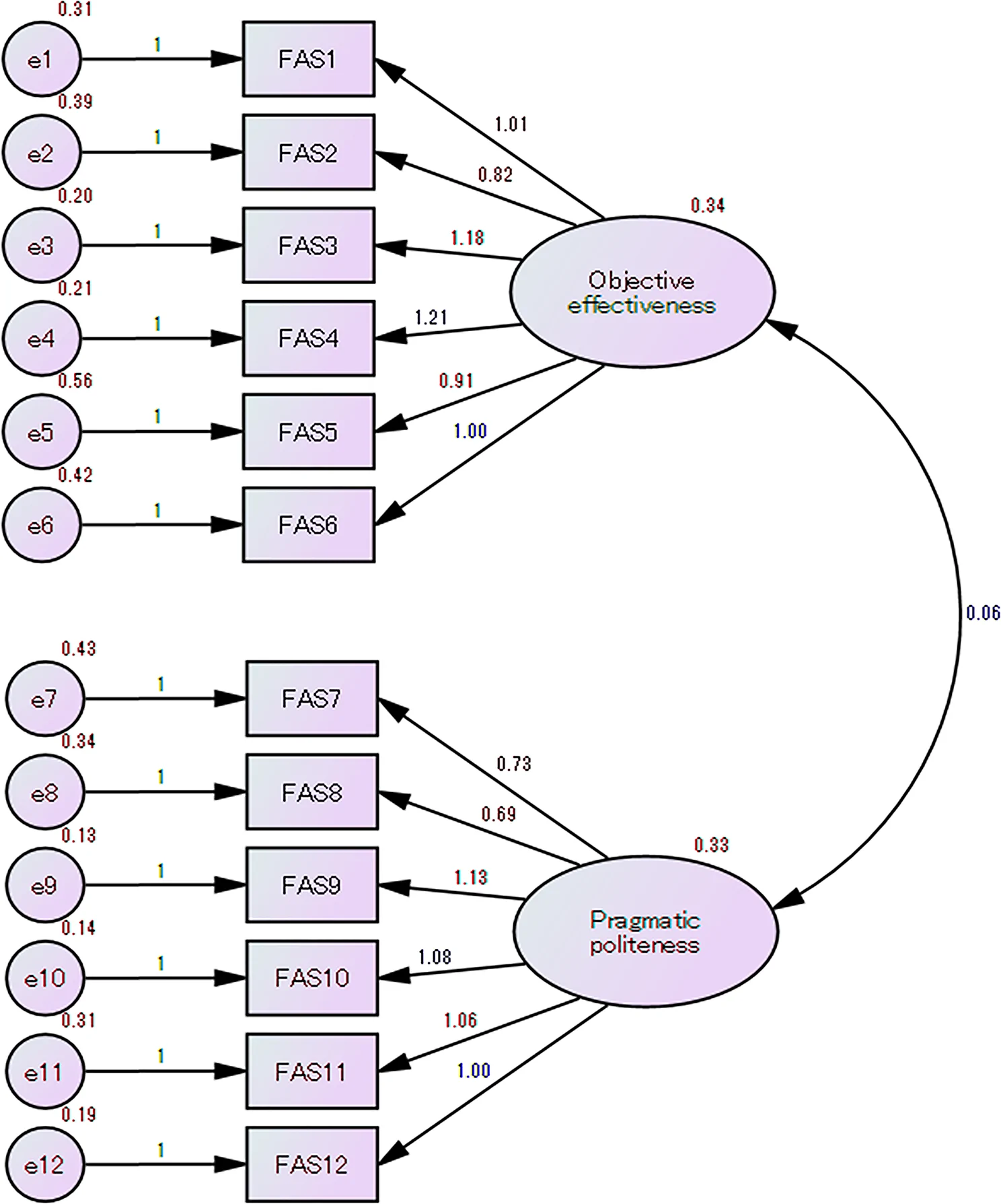
Path diagrams of the confirmatory factor analysis used to assess the structural validity. Squares represent the observed variable (Functional assertiveness [FAS] item 1–12), and ellipses represent the subscales

The CFA using the full dataset (*n* = 415) was conducted to evaluate the fit of this two-factor model. Figure 1 shows the path diagram of CFA used to evaluate the structural validity of the FAS. The CFA results were as follows: CFI = 0.893, TLI = 0.867, SRMR = 0.048, RMSEA = 0.110. The CR and AVE values for the subscales were 0.86 and 0.52 for OE, and 0.87 and 0.54 for PP, respectively (Table 3). Additionally, a split-sample cross-validation was conducted as a supplementary analysis (Supplementary Tables 1 and Supplementary Figure 1, respectively). EFA of the first subsample (*n* = 196) supported a two-factor structure. CFA of the second subsample (*n* = 219) yielded the following model fit indices: CFI = 0.891, TLI = 0.864, SRMR = 0.052, and RMSEA = 0.115.

Single-headed arrows from the ellipses to the squares represent factor loadings, the single-headed arrow below each square represents residuals, and the double-headed arrow represents factor correlations.

#### Reliability

The reliability of the FAS was examined based on internal consistency (Table 3). Cronbach’s alpha coefficients were calculated for the total and each subscale, which ranged from 0.85 to 0.87, showing good internal consistency. To determine the correlation between item scores and subscale scores, the item-total correlation coefficients ranged from 0.57 to 0.76, indicating that internal consistency was recognized.

#### Criterion-related validity

To verify criterion-related validity, we compared correlations with an assertiveness evaluation scale developed for pharmacists and compared score distributions based on participation in assertiveness training programs and participation in interprofessional care conferences (Table 4). Significant positive correlations were observed between IAS-AS score and FAS total score (rho = 0.34, *p* < 0.01) and OE subscale score (rho = 0.35, *p* < 0.01). However, no significant correlation exceeding the pre-set threshold (rho > 0.2) was observed for the PP subscale (rho = 0.16, *p* < 0.01). Although participation in assertiveness training resulted in significant differences in the total FAS score and OE score distribution (*p* = 0.04, *p* = 0.01, respectively), no significant difference was found in PP score (*p* = 0.75). No significant differences were observed in the score distribution for either the FAS total score or the subscale scores for participation in interprofessional care conferences (*p* = 0.56, *p* = 0.98, and *p* = 0.20, respectively).

**Table 4:** Criterion validation (n = 415)

	FAS	*p*-value	OE	*p*-value	PP	*p*-value
IAS-AS score^a^	rho = 0.34	< 0.01	rho = 0.35	< 0.01	rho = 0.16	< 0.01
Participation in assertiveness training^b^						
Yes (*n* = 91), Median (IQR)	38.0 (34.0–41.0)	0.04	18.0 (15.0–21.0)	0.01	20.0 (18.0–22.0)	0.75
No (*n* = 324), Median (IQR)	36.0 (33.0–40.0)		17.5 (14.0–19.0)		19.0 (18.0–22.0)	
Participation in interprofessional care conference^b^						
Yes (*n* = 247), Median (IQR)	36.0 (33.0–41.0)	0.56	18.0 (14.0–19.0)	0.98	19.0 (18.0–22.0)	0.20
No (*n* = 168), Median (IQR)	37.0 (34.0–41.0)		18.0 (14.0–20.0)		20.0 (18.0-22.8)	

^a^Spearman’s rank correlation

^b^Mann–Whitney U test

AS, Assertive self-expression; FAS, Functional assertiveness scale; IQR, Interquatile range; OE, Objective effectiveness; PP, Pragmatic politeness

## Discussion

This study aimed to validate the FAS and investigate its reliability and applicability among community pharmacists in Japan. To the best of our knowledge, this is the first study to examine the psychometric properties of the FAS among community pharmacists. Our findings suggest the applicability of a two-factor structure and demonstrate good internal consistency, providing preliminary support for criterion-related validity. These findings suggest that the FAS is a reliable and valid tool with demonstrated applicability for measuring functional assertiveness among community pharmacists in Japan.

The EFA conducted in this study confirmed the two-factor structure of the FAS, and both the overall scale and each subscale demonstrated good internal consistency. However, the results of the CFA did not satisfy the preset criteria for CFI, TLI, and RMSEA. Nevertheless, the SRMR yielded a favorable value of 0.048 (< 0.08), suggesting a certain degree of applicability. EFA is an exploratory approach that extracts structures from data, whereas CFA strictly evaluates fit to theoretical models derived from prior research. Hopwood and Donnellan reported that when measuring complex concepts, such as personality scales, evaluations should not rely solely on strict CFA cutoff values but should instead assess models while considering their practical significance [34]. The wording of the FAS items may have influenced responses, as the definition of “teammates” may have varied between responders depending on whether they viewed pharmacy staff as workplace colleagues or as part of a broader community healthcare team that includes physicians and nurses. In particular, it has been reported that the Japanese cultural concept of “*uchi to soto*” (inside one’s group and outside it) influences assertive communication [35]. On the other hand, items 6 and 12 that used the term “Friends” and items 3 and 4 that used “Manners and Personal Appearance” are based on scenarios encountered in the daily lives of university students [21]. Thus, these contexts differ significantly from the specialized and clinically demanding clinical situations pharmacists encounter such as recommending prescriptions to physicians or coordinating opinions with colleagues and other professionals. Although the FAS was previously found to be applicable to parents of children with disabilities [22] and psychiatric patients [23], this study attempted to use it without modification. However, these findings suggest that the current items do not fully capture the realities of community pharmacist practice, where ensuring medication safety is a primary concern. It is necessary to optimize the item content in future research to incorporate the specific scenarios and interpersonal dynamics unique to the community pharmacy setting.

In this study, participation in an assertiveness training program was significantly associated with higher FAS and OE scores but not higher PP scores. Although this study did not evaluate the specific curriculum of the training program attended by the participants, the significant difference observed in the total scores suggests that FAS is appropriate for evaluating the overall effectiveness of educational interventions. Among the subscales, significant differences were observed only in OE scores. However, the reason for this discrepancy remains unclear due to the lack of detailed information about training content. Therefore, caution is required when interpreting subscale-level sensitivity to educational interventions. Further pre-post studies evaluating specific training curricula are necessary. In terms of the relationship with IAS-AS, a significant association was found with FAS. Previous studies have linked IAS-AS to actual prescription recommendation behaviors [18, 19], suggesting that FAS may also serve as a potential indicator of clinical behaviors. However, unlike IAS-AS, which primarily assesses attitudes toward assertive self-expression, FAS is specifically designed to capture actual, more nuanced communication. In clinical practice, community pharmacists must maintain relationships with physicians by balancing both assertive and non-assertive expressions to achieve their recommendation goals. Further research is needed to investigate the precise relationship between FAS scores and actual prescription recommendation outcomes. On the other hand, regarding participation in interprofessional care conferences as another context of collaboration, no significant association was found with FAS scores. This finding *c*ontrasts with a previous study that reported a positive relationship with IAS-AS [33]. This discrepancy may be explained by two factors. First, as an external factor, conference participation may have been influenced by corporate policies and institutional incentives for community pharmacies such as dispensing fees. Second, as a measurement factor, IAS-AS assesses the degree of assertive self-expression within multidisciplinary teams, whereas the FAS specifically evaluates the achievement of clear objectives, such as enhancing patient safety, while maintaining professional relationships. These results likely reflect the difference between simply expressing oneself openly at a conference and the strategic communication required to achieve clinical goals without compromising relationships with physicians. However, this study is limited to evaluating care conference attendance alone. Future research is needed that also assesses factors such as the frequency of care conference participation and the level of involvement.

This study has several limitations. First, there is a potential for selection bias. The average age of community pharmacists in Japan was 46.8 years old in 2024 [36]; however, the population of the present study is skewed toward a relatively younger age group (mean = 41.0 years old). Furthermore, the survey response rate (18.9%) was relatively low, and since the sample was selected from two pharmacy chains, it cannot be said to fully represent community pharmacists nationwide. The organizational culture characteristics of these two chains, as well as the voluntary self-selection of participants, may have led to the inclusion of a group with a stronger interest in interpersonal communication, causing homogenization of the responses. However, Cronbach’s alpha coefficients for FAS, OE, and PP remained within the optimal range of 0.85 to 0.87 and remained significantly below the threshold of 0.95, which is often indicative of serious response bias. These results suggest that any bias that was present did not lead to severe distortion that compromised the overall internal consistency or reliability of the scale. Moreover, since this study was conducted via an online survey, it is possible that individuals who were more comfortable using technology were more likely to participate. Caution is needed regarding the generalizability of the findings, and further verification through large-scale surveys is necessary. Second, this study did not assess reliability using test-retest reliability. Due to the anonymity of the survey and difficulties in ensuring a longitudinal sample size, it was not possible to conduct a second assessment. Since the FAS is a self-report assessment of usual interpersonal communication, we believe longitudinal evaluation is necessary to conduct a more robust reliability verification. Third, as this study is cross-sectional, the assessment of construct validity is considered limited. To enhance the robustness of construct validity, it is necessary to longitudinally verify whether changes in FAS scores predict subsequent changes in related behaviors or outcomes such as improvements in communication quality and achievement of specific challenges. Finally, the item content of the FAS may not fully capture the specific professional contexts of pharmacists, which likely influenced the model fit in the CFA. Although we conducted an independent split-sample cross-validation to ensure the robustness of the factor structure, the participating community pharmacists were recruited from only two specific institutions. Future research should address these limitations by modifying the FAS items to fit specific work contexts and conducting nationwide surveys that include verification of longitudinal retest reliability.

## Conclusions

This study evaluated the reliability and validity of the FAS among community pharmacists in Japan. The FAS demonstrated good internal consistency while maintaining a two-factor structure similar to the original version. The model fit in the CFA suggests that the current items, which were originally developed for university students, need significant optimization to align with the professional healthcare environment. Although the current scale assessed pharmacists’ functional assertiveness, some of the indicators did not fit the factor structure well. This underscores the importance of developing a revised version of FAS that is specifically tailored to pharmacy practice as an essential preliminary condition for its practical application in clinical settings. This modified FAS could potentially be utilized to assess communication training programs and support pharmacists in managing work-related interpersonal stress, with possible benefits for interprofessional collaboration and patient care.

## Supplementary Information

Below is the link to the electronic supplementary material.

Supplementary Material 1

## Data Availability

The datasets used and analyzed during the current study are not publicly available because they contain information that could compromise the privacy of research participants. Deidentified datasets are available from the corresponding author on reasonable request.

## Abbreviations

AS: Assertive self-expression
AVE: Average variance extracted
CFA: Confirmatory factor analysis
CFI: Comparative fit indices
CR: Composite reliability
EFA: Exploratory factor analysis
FAS: Functional Assertiveness Scale
KMO: Kaiser-Meyer-Olkin
IAS: Interprofessional Assertiveness Scale
OE: Objective effectiveness
PP: Pragmatic politeness
RMSEA: Root mean square error of approximation
SRMR: Standardized root mean squared residual
TLI: Tucker-Lewis index
